# Comparison of the different animal modeling and therapy methods of premature ovarian failure in animal model

**DOI:** 10.1186/s13287-023-03333-4

**Published:** 2023-05-18

**Authors:** Fangfang Dai, Ruiqi Wang, Zhimin Deng, Dongyong Yang, Linlin Wang, Mali Wu, Wei Hu, Yanxiang Cheng

**Affiliations:** 1 Department of Obstetrics and Gynecology, Renmin Hospital of Wuhan University, Wuhan, 430060 Hubei China; 2 Department of Obstetrics and Gynecology Ultrasound, Renmin Hospital of Wuhan University, Wuhan, 430060 China

**Keywords:** Premature ovarian failure, Animal model, Hormone replacement therapy, Transplantation of stem cells, Chemotherapy drug

## Abstract

Incidence of premature ovarian failure (POF) is higher with the increase of the pace of life. The etiology of POF is very complex, which is closely related to genes, immune diseases, drugs, surgery, and psychological factors. Ideal animal models and evaluation indexes are essential for drug development and mechanism research. In our review, we firstly summarize the modeling methods of different POF animal models and compare their advantages and disadvantages. Recently, stem cells are widely studied for tumor treatment and tissue repair with low immunogenicity, high homing ability, high ability to divide and self-renew. Hence, we secondly reviewed recently published data on transplantation of stem cells in the POF animal model and analyzed the possible mechanism of their function. With the further insights of immunological and gene therapy, the combination of stem cells with other therapies should be actively explored to promote the treatment of POF in the future. Our article may provide guidance and insight for POF animal model selection and new drug development.

**Graphical Abstract:**

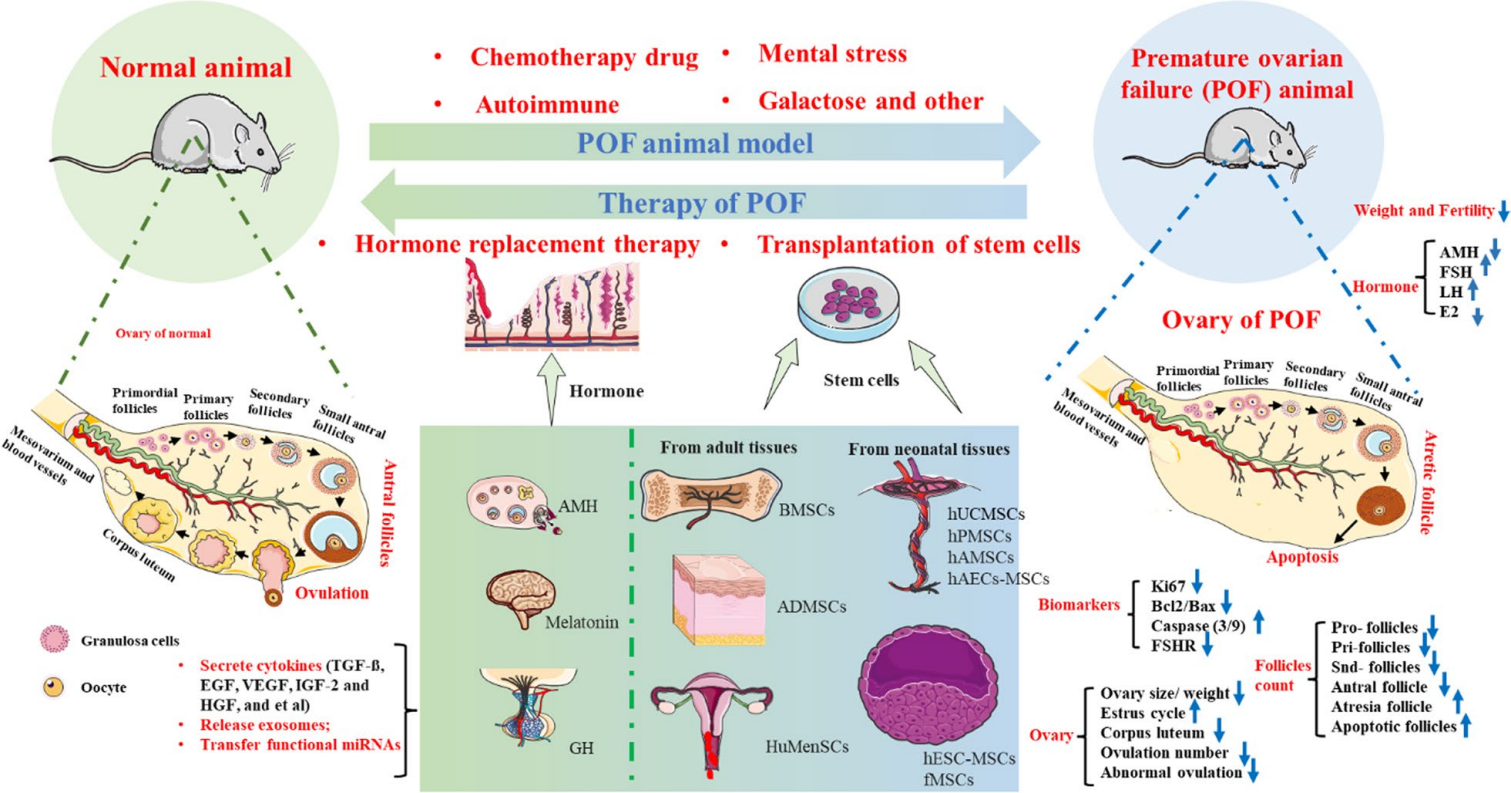

## Introduction

Premature menopause is closely associated with premature ovarian failure (POF), also known as premature insufficient ovarian failure (POI). The incidence of POF was 0.01% in women aged 20 years, 0.1% in women aged 30 years, and 1% in women aged 40 years [1]. The etiology of POF is very complex and closely related to genetics, immune diseases, drugs, surgery, and psychological factors [2]. Currently, according to the etiology of POF, the widely used POF animal models can be classified as chemotherapy drug-induced POF models, autoimmune POF models, POF models of mental stress, and galactose (GAL)-POF models. However, different POF animal model methods have their advantages and disadvantages. For example, the stability of POF animal models of autoimmune and mental stress is low. GAL-induced animal model can better simulate the physiological aging characteristics of clinical POF patients, but the success rate is lower. Hence, it is still a big challenge to select an ideal animal model for drug intervention and mechanism exploration.

Clinically, long-term hormone replacement therapy (HRT), mainly estrogen and progesterone, is the most common treatment for women with POF. The therapeutic and protective effects of some hormones, such as anti-Müllerian hormone (AMH), melatonin, and growth hormone (GH), have been confirmed and accepted. However, long-term use of HRT may increase the risk of cardiovascular disease and cancer [3]. So far, POF is still clinically irreversible. It is an urgent need to find advanced treatment strategies. Recently, the role of stem cells in the treatment of POF animal models has been gradually explored. Stem cells can differentiate into ovarian cells in the microenvironment of POF, to supplement the number of normal ovarian cells [4]. In addition, the regenerated ovarian cells by stem cells can secrete female hormone to maintain the hormone balance and improve women's symptom caused by the decline of ovarian function [5].

In our review, we firstly summarized different POF animal model methods and compared their advantages and disadvantages. Next, we summarized the recently published data on HRT and stem cells in the POF animal model. Our article may provide guidance and insight for POF animal model selection and new drug development.

## Comparison of different POF animal models

### POF animal model of chemotherapy drugs

It is estimated that more than 6.6 million women are diagnosed with cancer each year, approximately 10% of whom are aged younger than 40 years. The most important and common causes of POF are chemotherapy and radiation therapy in cancer treatment. Cytotoxic chemotherapy and radiotherapy have improved survival in many cases, but alterations in gonadal function are one of the most common long-term side effects of treatment. Some chemotherapeutic agents are associated with gonadal toxicity (e.g., cyclophosphamide (CTX), bucillamine, and nitrogen mustard), while others have minor or unquantifiable effects (e.g., doxorubicin (DOX), vinca alkaloid cisplatin (CIS), and nitrourea). An animal study showed that anticancer drugs (doxorubicin and paclitaxel) reduced the number of primitive and developing follicles in goat preantral follicles [6]. Other studies have also shown that the incidence of POF in women with breast cancer treated with docetaxel + pirarubicin + ifosfamide (DTC) chemotherapy is higher than that in healthy women [7]. Using CTX, methotrexate and fluorouracil (CMF) for more than 4 cycles increase the risk of infertility by more than 80% [8]. The pathophysiological mechanism of chemotherapy-associated POF is that the drugs destroy DNA, induce apoptosis of normal ovarian follicles, and block ovarian vascularization, thus interfering with the functional and structural features of oocytes [8, 9]. Recently, the pathogenesis of POF has been understood to some extent, but an appropriate animal model of POF will benefit the development of new drugs and observation of efficacy. At present, the most common drugs include CTX, tripterygium glycosides (TG), busulfan (BF), CIS, DOX, etc.

CTX inhibits cell proliferation because of its strong immunosuppressive effect. It significantly improves the clinical symptoms of patients with refractory nephropathy and has been widely used in the nephrology field. However, it is associated with serious side effects on female gonads, leading to ovarian damage, manifested as menstrual disorder, secondary amenorrhea, and even POF. The animal model of CTX-induced POF is simple in operation, is short in cycle, and only needs a single dose. TG exists a killing effect on rapidly proliferating cells (such as ovarian follicle cells), the mechanism of which mainly leads to DNA base mismatch and DNA chain rupture. The effect of DNA damage leads to a decrease in estrogen secretion in the ovary, which negatively increases FSH. The disadvantage of the TG-induced POF animal model is that the modeling cycle is long, while the advantage is that oral administration is relatively safe compared with other drug models [10]. CIS induces ovarian injury through the interaction of various factors, including activating apoptosis and the oxidative stress response in ovarian cells. CIS inhibits cellular DNA replication and RNA transcription, arrests cells in G2 phase, and leads to apoptosis. CIS-induced POF animal model has a low cost, a short cycle, low mortality and shows histological and endocrine changes similar to clinical POF ovaries. DOX is an anti-tumor antibiotic that inhibits the synthesis of RNA and DNA, and has the strongest inhibitory effect on RNA. A single intraperitoneal injection of DOX into ICR mice (7.5 mg/kg) resulted in a significant reduction in ovarian size and weight one month after treatment [11]. However, this approach of the POF model has rarely been reported in other articles, it maybe that the effect of this acute injury model is unstable and uncertain.

Clinicially, chemotherapy drugs are widely used, and the damage caused by their toxicity cannot be ignored. How to balance the therapeutic effect and toxic reaction of chemotherapy drugs and improve the safety of clinical drugs are the primary problems. Thus, seeking the prevention and treatment measures, and exploring the occurrence and development mechanism of diseases are beneficial. Animal model studies are helpful to understand the occurrence and development of human diseases more conveniently and effectively.

### The autoimmune POF animal model

Immune factors account for 10–30% of POF, which may be simple ovarian autoimmune disease or accompany by other immune diseases. Recent studies have shown that the risk of POF in women of childbearing age with autoimmune diseases is increasing. Early diagnosis of immune POF patients is challenging, and these patients are often in a state of ovarian failure at the time of treatment.

ZP3 glycoprotein is a critical zona pellucida glycoprotein and sperm receptor. Immunizing animals with ZP3 glycoprotein can cause ovaritis by activating T cells, and IgG antibodies against human recombinant ZP3 can lead to follicle destruction [12]. The method of inducing  immunocompromised POF mice with ZP3 glycoprotein is easy to establish, with a short cycle, high survival rate of mice (100%), and high success rate (80–90%). In addition, the mouse ZP3 protein shares 67% homology with human ZP3. ZP3 polypeptide induces mice to produce ZP3 polypeptide antibody, which binds to ovarian ZP3 to cause an immune response and interferes with information exchange between oocytes and granulosa cells. The above can induce ovarian atrophy, anovulation, and other manifestations, such as human POF. Hence, the ovarian histomorphology of the ZP3 polypeptide-induced POF mouse model is similar to that of human POF autoimmune ovaritis [12]. The ZP3 glycoprotein-induced POF mouse model is a classical modeling method to explore the pathogenesis and pathological changes of autoimmune POF.

Another method of antoimmune POF animal is that the supernatant protein of ovarian tissue of rats combined with Freund's adjuvant serves as an ovarian antigen. The rat model of autoimmune POF was successfully established by subcutaneous injection of 0.35 mL of ovarian antigen three times every 10 days [13]. Because this method is rarely used, it remains unclear what concentration of ovarian antigen can be used to successfully construct an autoimmune POF model. Moreover, studies have shown that removing the thymus of 3-day-old neonatal mice induces autoimmune ovaritis and leads to complete oocyte loss in adult mice [14]. However, thymectomy for newborn mice is difficult to perform and has a high mortality rate.

### The POF animal model of mental stress

Psychological stress, for example chronic anxiety, sadness, fear, and other negative emotions, can lead to POF by altering the function of the hypothalamic–pituitary–target gland axis, leading to the appearance of hypothalamic–pituitary–ovarian axis disorder. The failure of the feedback regulation of the hypothalamic–pituitary–ovarian axis disrupts the balance of the neuroendocrine–immune biomolecular network and ultimately leads to POF. Stress POF animal model can be constructed by alternately administering different frequencies of sound-light-electricity stimulation [15]. The decrease in biomolecules in the hypothalamic–pituitary–adrenal axis led to a significant decrease in biomolecules in the hypothalamus (β-EP, IL-1, NOS and GnRH), leading to a hormone decrease in the target gland layer (E2) in the pituitary–ovarian axis; the pituitary layer (FSH and LH) showed little change [15]. A chronic unpredictable mild stress (CUMS) model was constructed by alternating daily fasting and water deprivation, forced swimming, noise interference, and plantar electrical stimulation for 35 days. The results suggested that the CUMS rat model exhibited depression-like behaviors. CUMS causes psychological stress and decrease ovarian reserve in female rats [16]. The advantages of this modeling approach are that it is consistent with known major causative agents of human POF, and pathogenic pathways and pathological changes are similar to clinical observations.

### Galactose and other POF animal models

Galactosemia (GAL) is an autosomal recessive genetic disease caused by the deficiency of galactosidase in the body, resulting in the obstruction of the stereo isomerization process of GAL. Because of the accumulation of GAL and its metabolites in the body, patients eventually develop liver, kidney, eye, nervous, and reproductive system damage. The main clinical manifestation of reproductive system injury is POF or primary amenorrhea. The main mechanism is as follows: the level of GAL in the cell is increased, which is catalyzed by aldose reductase and then is converted to galactol. However, the cell cannot further metabolize GAL, so GAL accumulates in the cell and affects the normal osmotic pressure, causing cell dysfunction. However, the increase in GAL concentration will cause direct damage to granulosa cells, oocytes, and follicular membrane cells of ovarian tissue, while the metabolites of GAL will cause parenchymal damage to the ovary [17]. The offspring of pregnant rats feeding with GAL from Day 3 to Day 21 postpartum showed different manifestations of ovarian dysfunction when they were adults [18]. The model had a success rate of approximately 63%. Aging is the most common cause of POF. D-GAL can accelerate the ageing process, which is similar to observations of normal ageing processes. Hence, the D-GAL-induced model is widely used in aging-induced POF animal model studies [18]. Approximately 75–96% of women have galactosamic gonadal dysfunction. This is because GAL awakens the biological activity of FSH and produces direct ototoxic effects [19]. POF animal model can be successfully constructed by subcutaneously with D-Gal (200 mg/kg/d) daily for 42 days [20]. The process of establishing the model is simple, and the time required is short.

Overall, the chemotherapy drug induced model is a classic and simple animal model for studying POF. However, there still exist many disadvantages. For example, CTX-induced POF animal model may exist many side effects, including myelosuppression and bleeding [21]. TG-induced POF animal model needs long molding time [10]. CIS-induced POF animal model is too toxic and may lead to death [22]. The success rate of DOX-induced POF animal model is uncertain [11]. The autoimmunity-induced model is most related to the etiology of human POF, but the operation of model is relatively complicated. Thymectomy for newborn mice is difficult to operate and has a high mortality rate [14]. The psychological factor-induced model is consistent with the pathogenesis factors of POF. But the model construction time is long, and the stability of this kind of model must be further determined [15]. Although the GAL animal model can better simulate the physiological aging characteristics of clinical POF patients, this model has a lower success rate and longer cycle [18]. Table 1 systematically compares the advantages and disadvantages of different animal models of POF.

**Table 1:** The animal model of premature ovarian failure (POF)

Method of model	Animal	Method of administration	Dosage of administration	Advantages	Disadvantages	The model from References
CTX	Wistar rat (180–200 g)	Ip: 14 days	1st: 50 mg/kg; 2–14 th: 8 mg/kg	The most common model; the operation is simple	Myelosuppression and bleeding	[21]
	SD rat (150 g)	Ip: 14 days	1st: 50 mg/kg; 4–15 th: 5 mg/kg			[23]
	SD rat (8 weeks)	Ip: 14 days	1st: 50 mg/kg; 2–14 th: 5 mg/kg			[24]
TG	SD rat (220–250 g)	Ig: 70 days	For 10 weeks: 40 mg/kg/days	High safety	Long molding time	[10]
CTX + BF	Wistar rat (180–220 g)	Ip (CTX) + ih (BF)	1st: CTX:120 mg/kg + BF 12 mg/kg	Simple operation, short cycle, only a single dose	–	[25]
CIS	SD rat (180–250 g)	Ip: 6 days	2 mg/kg, Daily, 6 days	Low-cost, short cycles, low mortality	The lethal dose (50) was 7.4 mg/kg	[22]
	SD rat (320 ± 10 g)	Ip: 10 days	1.5 mg/kg, Daily, 10 days			[26]
DOX	ICR mice (7–8 weeks)	Ip: 7.5 mg/kg,	Single dose	The operation is simple	The model success rate is uncertain	[11]
ZP3 glycoproteins	BALB/c mice (18–22 g)	SI 1st and 14 th	1st: 0.16 mg/mouse 14th: 0.16 mg/mouse	Short cycle, high survival rate of mice (100%), and the high success rate (80–90%)	–	[27]
	BALB/c mice (7–8 weeks)	SI 1st and 14 th	1st: 0.16 mg/mouse 14th: 0.16 mg/mouse			[28]
OA + FIA	SD rat (8 weeks)	SI	3 times, once every 10 days. OA:FIA = 1: 1	–	This method is rarely used	[13]
Thymus removing	BALB/c mice	Surgery	Removing the thymus of 3-day-old neonatal mice	90% developed autoimmune ovaritis and POF	Thymectomy for newborn mice is difficult to operate and has a high mortality rate	[14]
Sound-light-electricity stimulation	SD rat (200–220 g)	For 20 days, 5 times per day	The acousto, optical, and electrical stimuli for 20 days	it is consistent with known major causative agents of human POF, and pathogenic pathways and pathological changes are like clinical observations	The success rate of the model is low and large samples are needed	[15]
CUMS	Wistar (~ 200 g)	For 35 days	Alternating daily fasting and water deprivation, forced swimming, noise interference, and plantar electrical stimulation			[16]
GAL	SD rat (Born 35 days)	Food pellet: 19 days	Food pellet with 35% galactose: from 3 days of conception continuing through weaning of the litters (21 days), the adult offspring were POF	Success rate:63%	The period is relatively long	[18]
D-GAL	Mice (7–8 weeks )	SI: for 42 days	Daily with d-gal (200 mg/kg/day)	The process of establishing the model is simple		[20]

*CTX* cyclophosphamide, *TG* tripterygium glycosides, *BF* busulfan, *CIS* cisplatinum, *DOX* doxorubicin, *ZP3* zona pellucida 3, *OA* ovarian antigen, *FIA*: Freund's incomplete adjuvant, *CUMS* constructed chronic unpredictable mild stress, *GAL* galactose, *D-GAL* d-Galactose, *Ip* intraperitoneal injection, *ih* hypodermic injection, *ig* intragastric administration, *SI* subcutaneous injection

## Common evaluation indicators of the POF animal model

Reasonable biological indicators are the key to judging the success of model construction and drug treatment effects. According to CALAS, the identification of experimental animal models requires the evaluation of their overall behavioral characteristics, tissues, organs, cells and molecules [29]. The evaluation of POF animal models mainly includes fertility, hormone, ovary and follicle, and biomarker evaluation.

### Fertility in the POF animal model

POF is a fertility decline disease caused by decreased ovarian reserve function, so the most intuitive detection method for animal models is the number of litters produced. In the animal model of POF, reproductive capacity is significantly reduced, including the fertility index (fraction of females that delivered offspring/total females), number of pups, and mean body weight of pups [30]. The drug-induced POF animal model takes advantage of the side effects of chemotherapy drugs on ovarian tissues. In addition to acute damage to the ovaries, toxic side effects on other organs from chemotherapy drugs can markedly affect the survival status of rats. Hence, the rat weight sharply declines [31].

### Histological assessment of the ovarian reserve in the POF animal model

The ovarian reserve refers to primordial follicles in the ovarian cortex of human females. Ovarian reserve tests are performed by directly or indirectly assessing the decline in the number of follicles [32]. Ovarian reserve of histological assessment includes the ovary volume and weight, the number of corpuses luteum, the length of the estrous cycle, the follicle count, the ratio of the ovulation number and abnormal ovulation [30]. In the ovarian tissues of the POF animal model, the ovarian volume and weight are decreased. Moreover, the corpus luteum, the ratio of the ovulation number and abnormal ovulation are lower, and the estrous cycle is extended. In addition, Pro-follicles, Pri-follicles, Snd-follicles, and antral follicles are reduced. However, atresia and apoptotic follicles are increased [33].

### Endocrine aspects of the ovarian reserve in the POF animal model

Endocrine levels are indirect reflections of decreased ovarian reserve function in POF. Clinically, measurement of AMH levels is useful in assessing the reserve of follicles and may be useful in assessing fertility potential. The lower ofAMH represents a decrease in ovarian reserve function [34]. The main role of FSH is to promote the growth and development of follicles and estrogen secretion. In addition, it can be used to identify the physiological condition of the female ovary. Clinically, an abnormally high value of FSH may indicates POF. LH and E2 can also be used as diagnostic criteria for POF. In the hormone secretion of the POF animal model, AMH and E2 are decreased, while FSH and LH are increased [35].

### Biomarkers of granulosa cells in the POF animal model

Most studies claim that ovarian apoptosis caused by oxidative stress and mitochondrial damage is the main cause of POF [30]. In the POF animal model, the expression of Ki67, Bcl2/Bax, and Caspase 3/9 is often used to measure ovarian proliferation and apoptosis levels. Follicle-stimulating hormone receptor (FSHR), a G protein-coupled receptor that binds to FSH, activates many intracellular signaling pathways, playing an important role in female follicle development and estradiol production. The gene mutation and downregulation of FSHR cause POI by preventing follicle development [36].

Overall, in the POF animal model, short-term measures of ovarian reserve function included a reduction in antral/atretic follicles and luteinization, disorder of the estrous cycle and hormone levels, and an increase in apoptotic biomarker expression. The long-term indicators were the decline in the fertility index and number of pups. Table 2 summarizes the common evaluation index of the POF animal model.

**Table 2:** Common evaluation index of different POF animal models

POF model	Body weight	Hormone				Biomarkers of granulosa cells					Fertility	The model from references
		AMH	FSH	LH	E2	Ki67	Bcl2	Bax	Caspase (3/9)	FSHR	Number of litters produced	
CTX	/	↓	↑	↑	↓	↓	↓	↑	↑	↓	↓	[30, 33]
VCD	↓	↓	/	/	↑/↓	/	/	/	/	/	/	[31]
CIS	ns	/	/	/	↓	/	/	/	↑	/	/	[22]
DOX	**/**	/	/	/	/	/	/	/	↑	/	/	[11]
GAL	**/**	/	↑	/	↓	/	/	/	/	/	/	[18]
pZP3	↓	↓	↑	↑	↓	/	/	/	/	↓	/	[35]
OA + FIA	↓	↓	/	/	↓	/	↓	/	↑	↓	/	[13]
HFHS	/	/	/	/	↓	/	/	/	/	↓	/	[37]

POF model	Ovary						Follicles count						The model from references
	Ovary size	Ovary weight	Estrous cycle	Corpus luteum	Ovulation number	Abnormal ovulation	Pro-follicles	Pri-follicles	Snd-follicles	Antral follicle	Atresia follicle	Apoptotic follicles	
CTX	**/**	↓	-	↓	↓	↑	↓	↓	↓	↓	↑	↑	[30, 33]
VCD	↓	**/**	-	**/**	**/**	**/**	↓	↓	↓	**/**	↑	**/**	[31]
CIS	**/**	**/**	/	**/**	**/**	**/**	↓	↓	↓	↓	**/**	**/**	[22]
DOX	↓	↓	/	**/**	**/**	**/**	*ns*	*ns*	*ns*	*ns*	*ns*	*ns*	[11]
GAL	**/**	**/**	/	**/**	**/**	**/**	**/**	**/**	**/**	↓	**/**	**/**	[18]
pZP3	**/**	↓	-	**/**	**/**	**/**	↓	↓	**/**	↓	**/**	↑	[35]
OA + FIA	**/**	**/**	/	↓	**/**	**/**	↓	↓	↓	↓	↑	**/**	[13]
HFHS	**/**	**/**	/	**/**	**/**	**/**	**/**	**/**	**/**	↓	**/**	↑	[37]

*AMH* anti-Müllerian hormone, *FSH* follicle-stimulating hormone, *LH* luteinizing hormone, *E2* estrogen, *BCL2* B cell lymphoma 2, *BAX* Bcl-2 associated X protein, *VCD* 4-vinylcyclohexene dicyclic oxide, *pZP3* zona pellucida 3 peptides, *CTX* cyclophosphamide, *Pro-follicles* primordial follicles, *Pri-follicles* primary follicles, *Snd-follicles* secondary follicles. Galactose (GAL), HFHS: High-fat diet,↓:Down-regulation, ↑: Up-regulation, ns: No statistical difference, /: no report; -: Interruption

## Current study of POF therapy in an animal model

### HRT in the treatment of the POF animal model

AMH is a hormone secreted by granulosa cells in preantral follicles and small antral follicles of the ovary. Detection of AMH can determine the functional status of granulosa cells and number of follicles. It demonstrated that the recombinant AMH protein can increase primordial follicless, rescuing the fertility of a CTX-treated POF animal model. The protective mechanism of AMH on CTX-induced follicular loss may be related to autophagy [38].

Melatonin (*N*-acetyl-5-methoxytryptamine, honey), a hormone produced primarily by the pineal gland of the brain, can also be produced by peripheral reproductive tissue (the ovary, and the placenta). Many studies have shown that exogenous melatonin has protective effects on the nervous system, kidneys, lungs, testes, uterus, and ovaries [39, 40]. In ovarian tissues, as a free group purifier in follicles, melanin promotes egg maturation, embryo development, and luteinization of granuloma cells [41]. It is reported that intraperitoneal administration of melatonin (15 or 30 mg/kg) for 15 days can successfully rescue CIS-induced primordial follicle loss by inhibiting phosphorylation of PTEN/AKT/FOXO3a pathway components and preventing FOXO3a nuclear shuttling in primordial follicles [42]. Another study showed that melatonin (20 mg/kg/day) taken orally for 34 days can increase the number of primordial follicles and antral follicles, increase body and ovary weight, and enhance the level of AMH by attenuating the activation of SIRT1 signaling pathway [43].

As a member of the growth factor family, GH (a peptide hormone secreted by the anterior pituitary gland) plays a crucial role in regulating growth and development, the gonadal axis, metabolism, and the mental state. Using mouse recombinant mouse GH (rmGH) for CTX-induced POF can significantly reduced ovarian granulocyte injury and the number of atretic follicles, and significantly increased the number of mature oocytes. They confirmed that GH may promoted ovarian tissue repair and estrogen release by activating the Notch-1 signaling pathway in ovarian tissue [44]. Subsequently, it confirmed that GH possesses a protective effect on ovarian tissue in the CTX-induced POF rat model by directly or indirectly promoting the balance between oxidative stress and oxidative detoxification of cells [45].

Table 3 lists the recent research status of HRT in the POF animal model. Although the short-term effects of HRT on POF animal models are effective, the long-term effects on fertility remain unknown. Thus, HRT has been little studied in animal models of POF. However, clinically, the improvement of POF symptoms mostly depends on personalized hormone treatment, aiming to maximize efficacy and reduce the associated risks.

**Table 3:** Hormone replacement therapy (HRT) in the POF animal model

Hormone	POF model		Hormone replacement therapy (HRT)				The HRT from references
	Animal	Drug	Method	Treatment	Assessment	Mechanism	
AMH	Mice (6 weeks)	CTX (1st:150 mg/kg, 75 mg/kg once/weeks for 4 weeks)	0.5 mg/kg, once/weeks for 4 weeks	Ip	Increase primordial follicles and decrease growing follicles. Could not restore the estrous cycle, but could rescue the fertility of CTX-treated mice	Induces autophagy	[38]
Melatonin	CD-1 Mice (5 weeks )	CIS (2 mg/kg for 15 days)	15 or 30 mg/kg daily, for 15 days	Ip	Increase the number of primordial follicles and antral follicles Decrease granulosa cell apoptosis	Block the phosphorylation of PTEN/AKT/FOXO3a pathway	[42]
	Mice (6 weeks )	TG (50 mg/kg/days for 34 days)	20 mg/kg/day, from day 8 to day 42	Oral	Increase the number of primordial follicles and antral follicles Increase body and ovary weight; Increase AMH Improve the estrous cycle	Attenuates the activation of SIRT1 signaling	[43]
	BALB/c-nu mice (6 weeks)	CIS (5 mg/kg weekly for 3 weeks )	10 mg/kg daily for 3 weeks	Ip	reduces primordial ovarian follicles loss and depletion caused by CTX protects reproductive endocrine function from CIS damage effectively protects sexually mature protects granulosa cells from damage	DNA protection and antioxidant effects	[46]
GH	C57BL/6 (4–5 weeks )	CTX (70 mg/kg a single)	100 µL of rmGH daily for 12 days Low dose: 0.4 mg/kg; Medium dose: 0.8 mg/kg; High dose:1.6 mg/kg	iv	The number of atretic follicles was reduced in the medium- and high-dose groups Ovarian weight was increased in the medium- and high-dose groups Medium-dose group can reduce FSH level	Activates the expression of NotCH-1 signaling pathway factors	[44]
	Rats (6 weeks)	CTX (1st, 50 mg/kg, 8 mg/kg for 14 days)	100 µL of rmGH once a week for 28 days Low dose: 0.4 mg/kg; Medium dose: 0.8 mg/kg; High dose:1.6 mg/kg	ih	The high-dose group can increase the number of oocytes Ovarian weight was increased in the medium- and high-dose groups did not reverse hormone levels	Directly or indirectly promotes the balance between oxidative stress and oxidative detoxification of cells	[45]

*AMH* anti-Müllerian hormone, *GH* growth hormone, *rhGH* recombinant human PEGylated GH

### Stem cells in the treatment of the POF animal model

#### Stem cells from adult tissues in the treatment of the POF animal model

After using HRT for POF, the risk of cancer and cardiovascular disease is increased. Recently, stem cell therapy has become increasingly popular in POF studies. BMSCs are a member of the adult stem cell family with low immunogenicity and generally exist in the bone marrow microenvironment. BMSCs are isolated from bone marrow extract. Density gradient centrifugation is a common method of preparing BMSCs derived from bone marrow [47]. Under certain circumstances, BMSCs can renew and differentiate into different cells, such as bone, cartilage, and fat cells. Despite the low survival rate and limited differentiation potential of BMSCs after transplantation, cytokines secreted by the ovary can induce BMSCs to migrate to damaged tissues. In the ovarian microenvironment, BMSCs can inhibit inflammation, reduce OS, and regulate immunity to promote ovarian tissue repair by secreting cytokines (VEGF, HGF, IL-6) [48]. The specific mechanism of BMSCs in the treatment of POF has been fully described in this article [48]. However, the number of BMSCs is very limited, and the immunomodulatory properties of BMSCs vary among species. The aggressive procedure is painful for the patient and carries a risk of infection. In addition, their differentiation potential, number, and maximum lifespan significantly decrease with age. These factors greatly limit the clinical application of BMSCs.

ADMSCs have low immunogenicity and can secrete many important growth factors, cytokines, trophic factors, and regenerative factors. Compared with ADSCs from elderly donors, ADSCs from young donors showed a higher proliferation rate, and their differentiation ability still exists with age. Therefore, ADMSCs have advantages over BMSCs. ADSCs also maintain the potential to differentiate into cells of mesodermal origin. Their low immunogenicity makes them suitable for allogeneic transplantation and the treatment of drug-resistant immune diseases [49]. ADMSCs have the advantages of availability and repeatability in autologous cell repair and regeneration [50]. ADMSCs are usually derived from fat tissue during liposuction, lipoplasty, or isolated lipotomy procedures and are digested with collagenase, followed by centrifugation and washing [51]. ADMSCs from the inguinal subcutaneous fat of 6–8-week-old nondiabetic rats can be obtained. It also demonstrated that ADMSC transplantation can reduce the expression of Pannexin1 and Caspase3 molecules to play an anti-apoptotic role in the ovarian tissues of a POF animal model. ADSCs stopped growing at 11~12 subculture, and the number of ADSCs was lower than that of BMSCs. *Mazini *et al*.* compared the advantages and disadvantages of ADMSCs as well as the research status of their therapeutic application [52].

HuMenSCs can be isolated from menstrual blood*.* HuMenSCs are much easier to repair than other adult stem cells, possibly making them a potential clinical donor source. *Gargett *et al*.* first extracted HuMenSCs, which can differentiate into adipocytes, osteoblasts, and lung epithelial cells [53]. The therapeutic potential of HuMenSCs has been demonstrated in diabetes [54], myocardial infarction [55] and liver failure [56]. Human endometrial mesenchymal stem cells (ESCs) derived from menstrual blood have the characteristics of mesenchymal stem cells (MSCs). MSC surface markers (CD29, CD44, CD49f, CD90, CD105 and CD117) and ESC markers (Oct4 and SSEA3/4) were highly expressed on the HuMenSC surface [57]. It confirmed the differentiation of HuMenSCs into ovarian-like cells (especially GCs) by injecting HuMenSCs into CTX-induced POF rats through the tail vein [58]. However, the source of HuMenSCs in menstrual blood is limited, and there is a risk of infection.

BMSCs, ADMSCs and HuMenSCs are adult MSCs that have been extensively studied in POF animal models at present. Table 4 summarizes the research status and possible mechanisms of three types of stem cells and their exosomes in POF animal models. Its main advantages are low immunogenicity, strong homing ability and strong ability to split and self-renew. However, most of their extraction procedures are invasive and carry the risk of infection.

**Table 4:** The transplantation of stem cells from adult tissues in the POF animal model

Stem cell types	POF model		The transplantation of stem cells from adult tissues				Stem cell from references
	Animal	Drug	Stem Cells	Treatment	Main effects of stem cell on POF	Mechanism	
BMSCs	Rats (5 weeks)	CTX (1st, 50 mg/kg, 8 mg/kg for 14 days)	BMSCs (1 × 10^6^ cells) in 100μL PBS for 2w	iv	Increase E2 and AMH, decrease FSH level; Recover the estrous cycle; Increase the number of basal and sinus follicles	BMSC-derived exosome Mir-144-5p has a protective effect on the apoptosis of CTX-damaged OGCs	[23]
	Rats (3 weeks)	3.2 Gy radiotherapy (0.48 Gy/min)	BMSCs (2 × 10^6^ cells) in 150μL PBS	iv	Enhances ovarian follicles; Increases serum estradiol- and follicle-stimulating hormone levels; Restores fertility	Upregulates Wnt/β-catenin and Hippo signaling pathways	[59]
Exosome from BMSCs	Mice (6–7 weeks)	CIS (5 mg/kg)	BMSCs-Exos (125 μg dissolved in 100 μL PBS	iv	Inhibit OGCs apoptosis; Increase E2;	delivering miR-644-5p to granulosa cells to regulate p53 expression of cells	[60]
	Rats (5 weeks)	CTX (1st, 50 mg/kg, 8 mg/kg for 14 days )	BMSCs-Exos (150 μg dissolved in 100 μL PBS; every other day for 2 weeks)	iv	Decrease FSH and LH, increase AMH, E2; Inhibit OGCs apoptosis;	Exosomes miR-144-5p inactivated the PI3K/AKT pathway by suppressing PTEN targeting	[23]
ADMSCs	Rats	CTX (120 mg/kg)	ADMSCs (1 × 10^6^ cells, passages 3–4)	Ip	Increase the number of primordial follicles and decrease the number of atretic follicles; Increase AMH level; Inhibit the apoptosis of follicle	Regulate the expression of Connexin43 and pAnnexin1	[61]
Exosome from ADMSCs	Mice (7–8 weeks)	CTX (120 mg/kg for 2 weeks)	hADSC-Exos (1 × 10^6^ cells, cocultured with hGCs)	Ip	Attenuate ovary damage; Increase the number of follicles; Enhanced the E2 and AMH levels and decreased the FSH levels; Inhibit OGCs apoptosis	Increase expression of SMAD2, SMAD3, and SMAD5 in vivo and in vitro	[62]
HuMenSCs	Mice (7–8 weeks)	CIS (2 mg/kg for 7 days )	HuMenSCs (2 × 10^6^/mL) in 200 μL PBS	iv	Increase body and ovary weight; Increase the number of follicles; Reduce OGCs apoptosis; repair ovarian injury, Stimulate regeneration, and improve ovarian function	Protects damaged ovaries by secreting FGF2	[63]
	Rats (8 weeks)	BF (36 mg/kg)	HuMenSCs (Passages 3)	iv	Improve follicle development; Promote AMH and E2 secretion	NO Report	[64]
	Rats (6–8 weeks)	BF (36 mg/kg)	HuMenSCs (1 × 10^6^ cells per 200 μL) in 1 mL PBS	iv	Increased body and ovary weight; Increase the number of follicles; Reduce OGCs apoptosis	NO Report	[58]
	Mice (18–19 g)	CTX (120 mg/kg) + BF (30 mg/kg)	hEnSCs (2 × 10^6^ cells, passages 5) in 20 μL PBS	Orthotopically inject	Increased body weight; Improved estrous cycles; Restored fertility	Reduce chemotherapy-induced depletion of the germline stem cell pool	[57]

*BMSCs* bone marrow mesenchymal stem cells, *ADMSCs* adipose-derived mesenchymal stem cells, *HuMenSCs* human menstrual-derived stem cells

#### Stem cells from neonatal tissues in the treatment of the POF animal model

Compared with adult tissue stem cells, human–neonatal tissue stem cells have lower immunogenicity, fewer ethical issues, a lower risk of infection, and a painless and noninvasive harvesting process and are easy to expand in vitro. Neonatal tissues such as the umbilical cord, placenta, amniotic membrane, or chorionic membrane can be obtained directly after delivery, avoiding invasive procedures and ethical concerns [65]. Moreover, MSCs isolated from these neonatal tissues represent ontogenetic younger cells, at least as attractive candidates for tissue engineering and regenerative medicine. hUCMSCs are the most widely studied MSCs in human–neonatal tissue stem cells and are mainly extracted from different compartments of the human umbilical cord. Compared with BMSCs, hUCMSCs have extensive advantages. On the one hand, the extraction process is noninvasive, preventing the risk of infection. On the other hand, hUCMSCs show higher proliferation and differentiation activity and faster self-renewal. hUCMSCs maintained a stable doubling time (DT) until the 10th generation, and BMSCs showed notably increased DT after only the 6th generation. hUCMSCs have been widely investigated in clinical therapeutic phase I or II trials, such as spinal cord injury, Alzheimer’s disease, and liver failure [66]. In recent years, hUCMSCs have received much attention due to their enormous therapeutic potential in POF therapy. Several studies have shown that the injection of hUCMSCs (1 × 10^6^/mL in 100 μL of PBS) through the tail vein can effectively improve the ovarian status. The method of extracting hUCMSCs from the human umbilical cord is fast, painless, and low immunity. However, there are more moral and ethical issues. The research progress of hUCMSCs in the POF animal model has been detailedly reviewed [4].

HESC-MSCs are cells isolated from an early embryo (before the gastrula stage) or primitive gonad. Compared with other sources of MSCs, hESC-MSCs,  they have a higher ability to proliferate and inhibit leukocyte growth [67]. HESC-MSCs show stronger anti-inflammatory properties than BMSCs [68]. HESC-MSCs can also overcome the obstacles encountered in harvesting MSCs from adult tissues, including the lack of appropriate donors, limited number of cells obtained in the acquisition process, limited ability to expand in vitro, and invasive nature of the procedure. HESC-MSCs have been shown to ameliorate chronic liver injury and autoimmune encephalitis. *Bahrehbar *et al*.* successfully extracted hESC-MSCs from the placenta and further confirmed that hESC-MSC transplantation was similar to BM-MSC transplantation, which can restore the structure and function of damaged ovarian tissue in CTX-induced POF mice and rescue fertility [69]. hESC-MSC transplantation has long been a controversial area. Proponents argue that it can help cure many intractable diseases because hESC-MSCs can differentiate into multifunctional APSCs, which most closely resemble human development. Opponents argue that hESC-MSC transplantation requires the destruction of embryos, which is anti-bioethical.

HPMSCs contain several stem cells based on placental anatomy: chorionic villi (CV-MSCs), amniotic membrane (AM-MSCs), chorionic plate (CP-MSCs), and umbilical cord Wharton Jelly (WJ-MSCs) [70]. Under the appropriate induction conditions, these placenta-derived MSCs can differentiate into various cell types. Compared with other stem cells from neonatal tissues, hESC-MSCs have the advantages of a convenient source, sufficient cell number, and easy isolation, culture, expansion, and purification, and they still possess the characteristics of stem cells after more than 30 generations. Transplantation of hESC-MSCs can restore the structure of damaged ovarian tissue and their function in CTX combined with BF-induced POF mice and rescue fertility. The possible mechanism is related to the promotion of follicle development, ovarian secretion, fertility, and ovarian cell survival through paracrine effects [69].

Human amniotic cells are divided into human amniotic epithelial cells (hAECs) and human amniotic mesenchymal stem cells (hAMSCs). Both cell types have the potential to differentiate into three layers of germ tissue. HAECs are a class of epithelial cells with stem cell characteristics that are not stem cells in nature because they cannot proliferate indefinitely. When hAECs were passaged to the fifth generation, the cells gradually became larger and older, and their proliferation ability was obviously weakened. However, hAMSCs could be transmitted to approximately the 30th generation without significant changes in cell morphology. hAMSCs had stronger differentiation and proliferation ability than hAECs. The biological characteristics of hAMSCs were superior to those of hAECs but were not superior in the expression of immune molecules. This effect may be because the cellular biological characteristics of hAMSCs, such as telomerase activity, expression level of pluripotent markers, cytokines, and collagen secretion, are superior to those of hAECs [71].

In addition to the above stem cells directly used in POF animal model therapy, other forms of stem cells have been investigated in POF treatment studies. Stem cell exosomes are a hot topic currently. Exosomes carry various microRNAs and proteins into target cells. Presently, exosomes from hUCMSCs and hAMSCs promote ovarian function by regulating the Hippo pathway and carry various microRNAs and proteins [72]. Collagen/hUCMSCs and Matrigel/hUCMSCs can also promote MSC adhesion and increase cell retention in the ovary [73]. In terms of the mode of administration in most animal studies, tail vein injections are the most widely used transplant method to deliver cells to recipients. However, most transplanted cells are trapped in the lungs and cannot reach the target organ. Hence, studies have designed sodium alginate-bioglass (SA-BG)-encapsulated hAECs to promote the adhesion properties, proliferative ability, migration, and homing ability of MSCs in the ovary [74]. Table 5 lists the transplantation of stem cells from neonatal tissues in the POF animal model.

**Table 5:** The transplantation of stem cells from neonatal tissues in the POF animal model

Stem cell type	POF model		The transplantation of stem cells from neonatal tissues				Stem cell from references
	Animal	Drug	Stem cells	Treatment	Main effects of stem cell on POF	Mechanism	
hUCMSCs	Rats (8 weeks)	CTX (1st, 200 mg/kg, 8 mg/kg for 14 days)	hUCMSCs (1 × 10^6^/mL) in 100 μL PBS	iv	Improve follicle development and hormone secretion; Reduce ovarian cells’ apoptosis	NO Report	[75]
	Mice (6–7 weeks)	CTX (120 mg/kg) + BF (30 mg/kg)	hUCMSCs (1 × 10^6^/mL) in 200 μL PBS	iv	Increase ovarian size and the number of primary and secondary follicles, decrease the number of atretic follicles; Increase E2 secretion and decrease FSH levels; Exert anti-apoptotic and anti-inflammatory effects	Activate AKT and P38 pathways; Exert anti-apoptotic and anti-inflammatory effects	[76]
	C57BL/6 (8 weeks)	ZP3	hUCMSCs (1 × 10^6^/mL)	iv	Increase serum E2, P, IL-4 levels, and decrease the levels of FSH, IFN-γ, IL-2; Increase the total number of follicles and decrease atretic follicles	Restored impaired ovarian and endothelial function mediated by changes in the Th1/Th2 cytokine ratio	[77]
	SD Rats (12 weeks)	CTX (1st , 200 mg/kg, 8 mg/kg for 14 days)	hUCMSCs (5 × 10^6^/mL) in 500 μL PBS	iv	Restore the disorder of hormone secretion (increase E2 and AMH); Restores follicle production and prevents the loss of secondary follicles); Prolong estrous; Improve pregnant rate and embryos numbers of POF rats	Improve ovarian failure via NGF/TrkA signaling pathway	[78]
	Wistar rats (180–220 g)	Paclitaxel (7.5 mg/kg for 1 weeks)	hUCMSCs (2 × 10^6^/mL) in 20 μL PBS	Orthotopically inject	Increase E2 and AMH, decrease FSH level; Increase antral follicle count	Regulate the tissue expression of CK 8/18, TGF-ß and PCNA; By directly triggering the ovarian epithelium and/or indirectly enriching the ovarian niche	[79]
	SD Rats (8 weeks)	Freund’s complete adjuvant	1 mL hUCMSCs with low (0.25 × 10^6^), medium (1 × 10^6^) and high (4 × 10^6^) doses	iv	Restore estrous cycle; Improve follicle development in rats; Increased serum E2, P4 and AMH; Reduce apoptotic granulomas and promote the proliferation of granuloma cells	Show dose-dependent effects on improving ovarian follicular development in POF rats	[13]
	C57BL/6 (6–8 weeks)	CTX (120 mg/kg) + BF (30 mg/kg)	hUCMSCs (1 × 10^6^/mL) in 100 μL PBS	iv	Increase levels of FSH and E2 secretion, Decrease follicular atresia, and increased the number of sinus follicles; Improve lymphocyte ratio	Improve ovarian function through PPAR and cholesterol metabolism pathways	[80]
	C57BL/6 (4–6 weeks)	CTX (70 mg/kg) + BF (12 mg/kg)	hUCMSCs (5 × 10^5^/mL) in 10 μL PBS	iv	Increase ovarian weight and follicle number, Decrease FSH, increase AMH, FSHR; Increase pregnancy rate	NO Report	[81]
Exosome from hUCMSCs	SD Rat (60–80 g)	CIS (3 μg/ml)	huMSC-EXOs (20 µg, 100 µg/ml	–	Alleviate apoptosis level Increase E2 level	NO Report	[82]
	C57BL/6–(8 weeks)	CTX (120 mg/kg two times)	huMSC-EXOs (20 μg/mL, 150 μg)	Ip	Improve the pregnancy rate (Exo 83.33% vs POF 33.33%) Increase FSHR promoted ovarian cells proliferation	Promoted ovarian granulosa cell (OGCs) Proliferation In Vitro by Regulating the Hippo Pathway and the Effect Was Inhibited by a YAP Inhibitor	[72]
	Wistar Rat (50–60 g)	CIS (4 µg/ml)	huMSC-EXOs (30 µg/ml	–	promote resistance to apoptosis and protect OGCs from CIS-induced injury in vitro	huMSC-EXOs could be incorporated into injured OGCs, accelerating the recovery of OGCs Exosomes carry a variety of microRNAs and proteins into target cells	[83]
Collagen/hUCMSCs	C57BL/6 (6 weeks)	CTX (40 mg/kg for 15 days)	Collagen/UCMSCs (2 × 10^5^/mL) in 10 μL PBS	Orthotopically inject	Increase E2 and AMH, decrease FSH level; Promote the formation of granulomatous cell, ovarian angiogenesis	Promote ovarian angiogenesis with the increase of CD31 expression	[73]
Matrigel/hUCMSCs	C57BL/6 (8 weeks)	CTX (1st , 100 mg/kg, 10 mg/kg for 14 days)	UCMSCs (5 × 10^5^/mL, passages 3–5) in 2.5 μL saline solution + 2.5 μL Matrigel	Orthotopically inject	Increase the number of follicles and decrease the rate of tissue fibro-degeneration; Increase the proliferation rate of granuloma cells; Increase the number of vascular radiosensitivity	Decrease the expression of TGFβ-1 Increase the expression of EGF, TGFβ-3 and VEGF-A	[84]
hESC-MSCs	C57BL/6 (6–8 weeks)	CTX (100 mg/kg) + BF (50 mg/kg)	hESC-MSCs (1 × 10^6^ cells)	iv	Promotes follicle development; Decrease FSH, increase AMH, E2; Restores fertility	Through paracrine VEGF, IGF-2 and HGF	[69]
	ICR or C57BL/DBA (7 weeks)	CIS (2 mg/kg for 10 days)	hESC-MSCs (passage 8~10)	iv	Increase the mean number of primary and primordial follicles, decrease the count of residual zona pellucida (a marker of apoptosis in ovarian follicles), Increase ovulation, embryo formation, and live birth rates	NO Report	[85]
hPMSCs	Balb/c (6–8 weeks)	ZP3	hPMSCs (1 × 10^6^ cells, passages 6)	iv	Increased E2 level and decreased FSH and LH levels; Increase follicles and decrease atretic follicles; Inhibit OGCs apoptosis	By ER stress IRE1α signaling pathway	[27]
	Balb/c (7–8 weeks)	ZP3	hPMSCs (1 × 10^6^ cells, passages 6)	iv	Improve Estrous Cycles; Inhibit Ovarian OGCs apoptosis; Increase E2 and FSH Secretion	Increase CD25^+^CD4^+^Treg cell, inflammatory regulations mediated by IFN-g and TGF-b	[28]
fMSCs	ICR (7–8 weeks)	CTX (120 mg/kg for 2 weeks)	fMSCs (1 × 10^6^ cells)	iv	Increased E2 and AMH level and decreased FSH levels; Increased sinus follicle number; Inhibit apoptosis	Regulate MT1, JNK1, PCNA and AMPK to reduce the oxidative damage of POI cells, enhance the oxidative protection and improve their anti-apoptosis effect	[86]
	ICR (4–6 weeks)	CTX (200 mg/kg) + BF (20 mg/kg)	fMSCs (5 × 10^5^ cells) in 5μL PBS	Orthotopically inject	Reduced apoptosis; Increase the number of primordial follicles and decrease the number of atretic follicles	Exosomal miR-10a derived from fMSCs protect the ovaries	[87]
hAMSCs	C57BL/6 (8 weeks)	Surgery (Hydrogen peroxide burns)	hAECs (1 × 10^6^ cells) in 300 μL PBS	Ip	Improve the estrous cycle; Decreased FSH levels; Increased the body weight and ovarian; Enhance the fertility Increase the number of primordial follicles and decrease the number of atretic follicles	Downregulate the expression of TNF-α and IL-1β	[88]
	C57BL/6 (8 weeks)	CTX (50 mg/kg for 15 days)	hAECs (2 × 10^6^ cells) in 200 μL PBS	iv	Increased E2 level and decreased FSH level; Increases the number of oocytes;	NO Report	[89]
Exosome from hAMSCs	C57BL/6 (10 week)	CTX (120 mg/kg for 2 weeks)	hAMSC-Exos (100 μL of PBS containing the 150 μg exosomes)	iv	Increased follicular numbers; Enhanced the E2 and AMH levels and decreased the FSH levels; Inhibit OGCs apoptosis	Inhibited the protein expression of SIRT4, ANT2, AMPK, and L-OPA1	[90]
hAECs	C57BL/6 (7–8 weeks)	CTX (120 mg/kg) + BF (30 mg/kg)	hAECs (12 × 10^6^ cells)	iv	Inhibition of chemotherapy-induced inflammation; Inhibit Ovarian OGCs apoptosis; Increase the number of cumulus oocyte complexes, increase secondary and mature follicles and decrease atretic follicles	Inhibit TNF-α-mediated cell apoptosis	[91]
	C57BL/6 (7–8 weeks)	CTX (120 mg/kg) + BF (30 mg/kg)	hAECs (2 × 10^4^ cells) in 10 μL PBS	Orthotopically inject	Increase secondary and mature follicles and decrease atretic follicles; Increase AMH, MVH, BMP15 and HAS2; Inhibit the apoptosis of primary human granulosa-lutein (hGL) cells; Promote angiogenesis and vasoformation	Activate TGF-β/Smad pathway in human luteinized OGCs	[92]
	ICR mice (7–8 weeks)	CTX (70 mg/kg for 1 weeks + 120 mg/kg for 1 weeks)	hAECs	Orthotopically inject	Increased E2 and AMH level and decreased FSH levels; Increased ovary weight; Increase secondary and mature follicles and decrease atretic follicles; Increase fertilizing ability; Increase the proliferation rate of OGCs	NO Report	[71]
Exosome from hAECs	Mice (7–8 weeks)	CTX (120 mg/kg) + BF (30 mg/kg)	hAECs Exosome (1st : 9th, orthotopic injection, 10 μL) and 2nd: 10th, tail vein injection, 100 μL))	Orthotopic injection, iv	Inhibit OGCs apoptosis; Protect the ovarian vasculature from damage; Maintain the number of primordial follicles	Transfer functional miRNAs, such as miR-1246	[93]
SA-BG encapsulated hAECs	Mice (8 weeks)	CTX (120 mg/kg) + BF (30 mg/kg)	hAECs (6 × 10^7^ cells) in 0.2 mL PBS + 2 mL SA-BG	Orthotopically inject	promoted the proliferation of granulomatous cells in antral follicles; Enhanced angiogenesis; Promoted the tube formation	Stimulated the secretion of pro-angiogenic factors	[74]

*hEnSCs* human endometrial mesenchymal stem cells, *hUCMSCs* human umbilical cord mesenchymal stem cells, *hESC-MSCs* human embryonic stem cell-derived MSCs, *hPMSCs* human placenta-derived mesenchymal stem cells, *hAMSCs* human amniotic mesenchymal stem cells, *hAECs* human amniotic epithelial cells, *fMSCs* fetal liver mesenchymal stem cell, *SA-BG* sodium alginate-bioglass

### Immunological and gene therapy in POF animal model

In recent years, advances in immunology and genome medicine have improved our understanding of the pathogenesis of POF [94]. An increased number of B cells, CD4^+^ T cells, Th17 cells, and a decreased CD8^+^ T cells, Treg cells in POF patients have been reported [95]. Besides, the cytokines (IL-1α, IL-2, IL-6, IL-8, TNF-α, IFN-γ, IL-17 and IL-21) are upregulated [95, 96], and IL-10 is downregulated in POF patients [97]. Based on these advances, many related treatments such as thymopentin (TP-5), Ab4B19, and prednisone have gradually become research hotspots. *Zhu *et al. demonstrated that thymopentin (TP-5) significantly reduces the proportion of activated T cells (CD3^+^/CD8^+^) and M1/M2 macrophages, and the expression of inflammatory factors was decreased [37]. Co-administration of mouse zona pellucida 3 (mZP3) protein in combination with a DNA vaccine encoding the mZP3 gene can meliorate autoimmune ovarian disease through inducing Treg cells and anti-inflammatory cytokine production [98]. A clinical prospective study showed that short-term treatment with a prednisone can increase serum E2 levels and improves follicle growth [99]. However, the study requires a larger sample size.

Currently, most gene therapy research of POF is limited to the cellular and animal levels. These genes, including NEAT1/miR-654, miR-146a, miRNA-190a-5p, miR-146b-5p, miR-133b, and TRERNA1, are transferred into cells to ameliorate the POF symptoms by inhibiting apoptosis of ovarian granulosa cells (OGCs), stimulating estrogen synthesis, increasing the number of normal follicles, and decreasing the number of atretic follicle (Table 6) [100–102]. Gene therapy is still in experimental stage; it is not sure that whether the treatment will have a positive effect on patients.

**Table 6:** The immunological and gene therapy in the POF animal model

Immune agents/genes	POF model		Immunological/gene therapy				Immunological/gene therapy from references
	Animal	Drug	Method	Treatment	Assessment	Mechanism	
TP-5	C57BL/6 mice (10 weeks)	HFHS (High-fat diet 8 g/kg + 200 μL of 30% high fructose syrup once a day via gavage) for 2 m	TP-5 (5 mg/kg) for 2 m	Ip	Decrease atretic follicles Increase ovary weight Increase peripheral blood E2 levels Improve lipid oxidative stress injury and blood lipids Attenuate proportion and activation of CD3+ T cells and type I macrophages	TP-5 upregulates BMP4/Smad9 signaling pathway to promote the balance and polarization of immune cell, and reduces the release of inflammatory factors and lipid oxidative stress injury	[37]
TrkB agonist antibody (Ab4B19)	C57BL/6 mice (6–8 w)	CTX (a single, 75 mg/kg, 200–300 μL) for 7 days	Ab4B19 (1 mg/kg), once every 4 days, for 16 days	iv	Promote oocyte maturation and follicle development Attenuate ovarian degradation Normalize gonadal hormone Inhibit apoptosis Enhance fertility	NO Report	[30]
pcD-mZP3 + mZP3 protein vaccine	C57BL/6 mice (8–10 weeks)	mZP3 (0.1 ml of 100 µg of CFA emulsified mZP3)	100 µg DNA and 100 µg protein vaccines per mouse	-	Ameliorate autoimmune ovarian disease Promote anti-inflammatory function Down-regulate the antigen-specific T-cell responses Induce adaptive Tr cells	The induction of the CD4^+^CD25^−^Foxp3^+^IL-10^+^ Treg cells suppress mZP3 antigen-specific T cell responses in vitro with decreasing the anti-inflammatory cytokine production	[98]
Prednisone	POF patients	–	25 mg four times per day for 2 weeks	–	2/11 patients demonstrated biochemical normalization, evidence of follicular growth by a rise of E2, and visualization on ultrasonography, and both spontaneously ovulated, conceived, had uneventful pregnancies, and delivered healthy children	NO Report	[99]
NEAT1/miR-654	C57BL/6 mice (8 weeks)	CTX (30 mg/kg every other day for 3 weeks)	–	Cell transfection	Eliminates the promoting effect of CTX on OGC apoptosis and autophagy	NEAT1 overexpression inhibits miR-654 and further regulates STC2/MAPK pathway	[103]
miR-146a	OGCs	–	80 nM miR‑146a inhibitor/wk	Cell transfection	Inhibit granulosa cell apoptosis	Attenuates the activation of miR‑146a/IRAK1/TRAF6/caspase‑8 signaling	[104]
miRNA-190a-5p	SD rats (12 weeks, 200 ± 20 g)	VCD (80 mg/kg/d for 15 days )	No treatment		Promotes primordial follicle hyperactivation	miRNA-190a-5p inhibits the expression of PHLPP1 and key proteins in the AKT-FOXO3a and AKT-LH/LHR pathways	[105]
miR-146b-5p	C57BL/6 mice (10 weeks)	HFHS diet (8 g/kg bodyweight + 400 μL of 30% d-glucose for 30 days )	400 μL of miR-146@ PLGA (20 mg/mL) once every 3 days	iv	Mitigates the HFHS-induced oxidative stress injury and aging in OGCs Increase ovary weight Increase the number of normal follicles, decrease the number of atretic follicle Increase the peripheral blood levels of estradiol, progesterone and 17α-hydroxy pregnenolone Decrease the peripheral blood levels of testosterone and dihydrotestosterone	miR-146b-5p overexpression attenuates the activation of the Dab2ip/Ask1/p38-Mapk signaling pathway and γH2A.X phosphorylation	[100]
miR-133b	ICR mice (21 days )	–	–	Cell transfection	Stimulates estrogen synthesis in OGCs	miR-133b down-regulates Foxl2 expression in OGCs by directly targeting the 30UTR, and inhibits the Foxl2-mediated transcriptional repression of StAR and CYP19A1	[101]
TRERNA1	KGN cells	–	10 mM TRERNA1 vector/10^7^ cells	Cell transfection	Inhibit KGN cells apoptosis	TRERNA1 may sponge miR-23a to suppress OGCs apoptosis in POF	[102]

*TP-5* thymopentin, *HFHS* high-fat diet, *TGs* tripterygium glycosides, *OGCs* ovarian granular cells, *VCD* 4-vinylcyclohexene dicyclic oxide, *HFHS* high-fat diet, *VCD* 4-vinylcyclohexene diepoxide

## Conclusion

Suitable and ideal POF animal models are essential carriers for drug development and mechanism research. Chemotherapy drug model is a classic animal model for studying POF. However, chemotherapy-induced POF animal model may exist many side effects, including myelosuppression and bleeding. POF animal model from autoimmunity and mental stress is the largest relationship with the etiology of POF, but the stability of the model needs to be further determined. GAL-induced animal model can better simulate the physiological aging characteristics of clinical POF patients, but the success rate is lower and the cycle time is longer. Hence, the study of the mechanism of POF and drug efficacy should select appropriate models according to the main purpose of the study. An ideal animal model would have the following characteristics: (1) the pathogenic pathways and processes like those observed in humans; (2) the pathological changes in the model can be reversed by drugs; (3) the reproducibility of the results [106]. In the future, a model of POF constructed by injection of chemotherapy drugs and GAL under ultrasound guidance may reduce side effects and improve model success rate. Besides, more efforts should be made to study aging-related POF. For example, constructing aging-induced animal model studies the role of MSCs and their exosomes in restoring ovarian function.

The breakthrough discovery of MSCs makes them an ideal source for POF therapy. Many animal and preclinical studies of MSCs for POF treatment have been conducted; the clinical application of MSCs has big challenges, including insufficient cell sourcing, immunogenicity, subculture, and ethical issues. In addition, the long-term survival and self-renewal of stem cells in ovarian tissue remain to be further studied. In the future, it is necessary to establish a professional quality inspection system of MSC production to ensure the functional potential and microbiological safety of MSCs. More importantly, multicenter, large-sample phase II or III trials are expected to confirm the therapeutic and safety effect of stem cells on POF rather than just POF animal models. Especially, it is also worth considering whether the regenerative properties of MSCs can stimulate tumor regeneration in the future. Exosomes is smaller, easier to produce, and can carry various microRNAs and proteins into target cells without risk of tumor formation [72]. Moreover, stem cell tissue engineering is also an effective strategy. Sodium alginate-bioglass (SA-BG)-encapsulated MSCs can support the survival of the transplanted cells at the initial phase of transplantation [74]. Moreover, the combination of stem cells with other therapies (such as gene and immunotherapy) should be actively explored to promote the treatment of POF in the future.

## Data Availability

Not applicable.

## Abbreviations

POF: Premature ovarian failure
POI: Premature insufficient ovarian failure
GAL: Galactose
AMH: Anti-Müllerian hormone
GH: Growth hormone
CTX: Cyclophosphamide
BF: Busulfan
CIS: Cisplatinum
DOX: Doxorubicin
TG: Tripterygium glycosides
FSH: Follicle-stimulating hormone
LH: Luteinizing hormone
E2: Estrogen
HRT: Hormone replacement therapy
ZP3: Zona pellucida 3
CUMS: Constructed chronic unpredictable mild stress
FSHR: Follicle-stimulating hormone receptor
rmGH: Recombinant mouse GH
MSCs: Mesenchymal stem cells
BMSCs: Bone marrow mesenchymal stem cells
ADMSCs: Adipose-derived mesenchymal stem cells
HuMenSCs: Human menstrual-derived stem cells
hEnSCs: Human endometrial mesenchymal stem cells
hUCMSCs: Human umbilical cord mesenchymal stem cells
hESC-MSCs: Human embryonic stem cell-derived MSCs
hPMSCs: Human placenta-derived mesenchymal stem cells
hAMSCs: Human amniotic mesenchymal stem cells
hAECs: Human amniotic epithelial cells
fMSCs: Fetal liver mesenchymal stem cells
TGF-β: Transforming growth factor-beta
VEGF: Vascular endothelial growth factor
EGF: Epidermal growth factor
IGF2: Insulin-like growth factor 2
HGF: Human growth factor
